# Differential Long Non-Coding RNA Expression Analysis in Chronic Non-Atrophic Gastritis, Gastric Mucosal Intraepithelial Neoplasia, and Gastric Cancer Tissues

**DOI:** 10.3389/fgene.2022.833857

**Published:** 2022-04-27

**Authors:** Xin-Yuan Liu, Tian-Qi Zhang, Qi Zhang, Jing Guo, Peng Zhang, Tao Mao, Zi-Bin Tian, Cui-Ping Zhang, Xiao-Yu Li

**Affiliations:** ^1^ Department of Gastroenterology, The Affiliated Hospital of Qingdao University, Qingdao, China; ^2^ Department of Gastroenterology, Qingdao Women and Children’s Hospital, Qingdao, China

**Keywords:** long non-coding RNAs, chronic non-atrophic gastritis, gastric mucosal intraepithelial neoplasia, gastric cancer, gene expression

## Abstract

Gastric cancer (GC) has a high incidence worldwide, and when detected, the majority of patients have already progressed to advanced stages. Long non-coding RNAs (lncRNAs) have a wide range of biological functions and affect tumor occurrence and development. However, the potential role of lncRNAs in GC diagnosis remains unclear. We selected five high-quality samples from each group of chronic non-atrophic gastritis, gastric mucosal intraepithelial neoplasia, and GC tissues for analysis. RNA-seq was used to screen the differentially expressed lncRNAs, and we identified 666 differentially expressed lncRNAs between the chronic non-atrophic gastritis and GC groups, 13 differentially expressed lncRNAs between the gastric mucosal intraepithelial neoplasia and GC groups, and 507 differentially expressed lncRNAs between the chronic non-atrophic gastritis and gastric mucosal intraepithelial neoplasia groups. We also identified six lncRNAs (lncRNA H19, LINC00895, lnc-SRGAP2C-16, lnc-HLA-C-2, lnc-APOC1-1, and lnc-B3GALT2-1) which not only differentially expressed between the chronic non-atrophic gastritis and GC groups, but also differentially expressed between the gastric mucosal intraepithelial neoplasia and GC groups. Furthermore, RT-qPCR was used to verify the differentially co-expressed lncRNAs. LncSEA was used to conduct a functional analysis of differentially expressed lncRNAs. We also predicted the target mRNAs of the differentially expressed lncRNAs through bioinformatics analysis and analyzed targeting correlations between three differentially co-expressed lncRNAs and mRNAs (lncRNA H19, LINC00895, and lnc-SRGAP2C-16). Gene Ontology and Kyoto Encyclopedia of Genes and Genomes databases were used to explore the functions of target mRNAs of differentially expressed lncRNAs. In conclusion, our study provides a novel perspective on the potential functions of differentially expressed lncRNAs in GC occurrence and development, indicating that the differentially expressed lncRNAs might be new biomarkers for early GC diagnosis.

## Introduction

According to global malignant tumor statistics released by the WHO in 2018, there are more than one million new cases of gastric cancer (GC) worldwide annually. GC ranks fifth in cancer incidence and is the third leading cause of cancer-related deaths (Ferlay et al., 2019). The incidence of GC in China is approximately six times that of the United States, which poses a serious threat to human life and health, and places a considerable burden on public health (Verma and Sharma, 2018). Due to the lack of specific clinical manifestations in the early stage of GC, more than 60% of patients have undergone local or distant metastasis when the diagnosis is confirmed, and the best treatment opportunity has been missed (Thrift and El-Serag, 2020). At present, GC diagnosis primarily relies on endoscopy, pathology, and serum tumor markers, such as carcinoembryonic antigen. The pathological results of gastroscopic biopsy are the gold standard for GC diagnosis, however, it remains difficult for certain patients to undergo early GC screening under endoscopy because of its high cost and invasiveness. Moreover, the effect of tumor markers is limited because of the lack of high specificity and sensitivity (Yang et al., 2016; Yu and Zheng, 2018). Therefore, it is crucial to investigate potential and effective biomarkers for early GC diagnosis.

Research on GC initially focused on the biological functions of protein-encoding genes. With the advancement of genome high-throughput sequencing technology, it has been revealed that <2% of the genome has the protein encoding function (Saw et al., 2021). The transcriptional products of the remaining 98% of genes are named non-coding RNAs (ncRNAs), which are involved as regulatory factors in the occurrence and development of many diseases (Wang et al., 2014; Booy et al., 2021). Long noncoding RNAs (lncRNAs) found in the nucleus and cytoplasm are novel ncRNAs. With the development of molecular biology technology, lncRNAs have become a research hotspot in the occurrence and progression of GC and other cancers (Luis et al., 2013). Although lncRNAs do not directly participate in the protein expression process, they can combine with RNA in several biological processes, such as gene expression regulation, cell cycle modulation, transcription process interference, and protein activation function (Sigova et al., 2013; Laurent et al., 2015). In recent years, various lncRNAs have been confirmed to be involved in the occurrence, invasion, metastasis, and prognosis of multiple cancers, including GC. For example, Endo et al. found that lncRNA HOTAIR was highly expressed in GC tissues and was positively correlated with tumor malignancy and poor prognosis (Endo et al., 2013). It has been reported that MALAT-1 might inhibit E-cadherin expression by regulating the epithelial-mesenchymal transition (EMT), thereby promoting the malignant proliferation of GC cells (Chen et al., 2017). Furthermore, the expression of lncRNA DANCR in GC tissues was significantly upregulated, which was correlated with tumor volume, clinical stage, lymph node metastasis, and depth of invasion. Simultaneously, it has been shown that SALL4 in GC cells could activate the expression of DANCR through the β-catenin pathway, thereby exerting a carcinogenic effect *in vivo* and *vitro* (Pan et al., 2018). LncRNA UCA1 was found to promote GC development by regulating the PI3K-Akt-mTOR axis (Li et al., 2017). lncRNA CCAT2 silencing could inhibit the PI3K/mTOR axis, further resist GC cell malignant development, induce autophagy, and initiate cancer cell apoptosis (Zhang et al., 2017; Yu et al., 2018). Gao et al. (2015) used the receiver operating characteristic curve to evaluate the sensitivity and specificity of UCA1 as a tumor marker, and discovered that plasma UCA1 had a high diagnostic value in GC. Various lncRNAs have been found to be closely related to GC and are expected to become molecular markers for early GC prediction. However, the functions and mechanisms of most lncRNAs have not yet been elucidated. In the future, a comprehensive and systematic exploration of differentially expressed lncRNAs will be required.

RNA sequencing (RNA-seq), also called transcriptome sequencing technology, is based on second-generation high-throughput sequencing technology to obtain almost all transcript expression information, including mRNA and ncRNA. This technology has the characteristics of high efficiency, low species restriction, and high coverage of the obtained information (Wang et al., 2009). In this study, high-quality samples were subjected to lncRNA library construction, comparison and splicing, and fragment screening after strict quality inspection of all samples, and then Illumina NovaSeq 6000 was used to sequence the lncRNA libraries. The data obtained were used for differential expression analysis of lncRNAs. We screened the differentially expressed lncRNAs between every two groups in chronic non-atrophic gastritis tissues, gastric mucosal low-grade/high-grade intraepithelial neoplasia tissues, and GC tissues using edgeR, and identified lncRNAs H19, LINC00895, lnc-SRGAP2C-16, lnc-B3GALT2-1, lnc-HLA-C-2, and lnc-APOC1-1 as research focus. Finally, we used RT-qPCR to verify the above six differentially expressed lncRNAs and explore new biomarkers for early GC diagnosis.

## Methods

### Sample Collection and Inclusion Criteria

All patients included in this study were treated with endoscopy or surgery at the Affiliated Hospital of Qingdao University from September 2019 to September 2020. The inclusion criteria were as follows: 1) all samples were confirmed by postoperative pathology; 2) no history of other tumors; 3) no tumor treatment was applied before surgery, such as radiotherapy, chemotherapy, or immunotherapy; 4) the histopathological classification diagnosis met the WHO diagnostic criteria for digestive system tumors (2019); and 5) all selected patients signed an informed consent form after the study was approved by the Ethics Committee of the Affiliated Hospital of Qingdao University.

We collected a total of 22 cases of chronic non-atrophic gastritis (gastritis group, gas1), 14 cases of gastric mucosal low-grade or high-grade intraepithelial neoplasia (gastric precancerous lesion group, gas2), of which 9 cases were of gastric mucosal low-grade intraepithelial neoplasia, 5 cases of gastric mucosal high-grade intraepithelial neoplasia, and 9 cases of GC (GC group, gas3). From the above three groups, five high-quality samples were screened to analyze the differentially expressed lncRNAs between every two groups and to explore lncRNAs with the same expression trend among the groups.

### RNA Extraction and lncRNA Library Construction

An RNA extraction kit (Aidlab, Beijing, China) was used to extract RNA from 45 patient tissue samples, which were diluted to a working concentration of 100 ng/ml and stored at −80°C. RNA (1 μl) was extracted and a NanoDrop 2000 spectrophotometer (Thermo Fisher Scientific, MA, United States) was used to conduct initial RNA sample inspection. Finally, 5 cases were selected from each group, and 15 samples that met the conditions were used for lncRNA library construction and sequencing analysis. The strand-specific library construction kit NEBNext® (NEB, MA, United States) was used for library construction, and the library was quantified using Qubit2.0 (Life Technologies, CA, United States). Agilent 2100 (Agilent Technologies, CA, United States) was used to detect the length of the inserted library. qPCR was used to quantify the effective concentration of the library. Different libraries that met the quality control standards were sequenced using the NovaSeq 6000 high-throughput sequencing platform (Illumina, CA, United States). LncRNA sequencing was conducted by Berry Hekang Biotechnology (Beijing, China).

### Screening of the Differentially Expressed lncRNAs

The original sequencing data were normalized and then compared with the human reference gene sequence using HISAT2 software (Pertea et al., 2016). StringTie software was used for transcript assembly (Pertea et al., 2016). Cuffmerge software was used to splice and concatenate the transcripts of each sample and obtain complete transcriptome information of the sequence (Trapnell et al., 2012). Subsequently, we used Cuffcompare software to remove transcripts that overlapped with the database annotated exon region and used Cuffquant software to select transcripts with a value of FPKM≥0.5. Moreover, CPC, CNCI, CPAT, PLEK, and PFAM software were used to analyze and identify non-coding transcripts (Kong et al., 2007; Mistry et al., 2007; Sun et al., 2013; Wang et al., 2013; Li et al., 2014; Finn et al., 2016). Then, the overlapping data of the software screening results were selected as the lncRNA datasets for analysis and prediction. Cuffdiff software was used to calculate the FPKM values of the transcripts (Trapnell et al., 2012). edgeR was used to perform a differential analysis of the transcripts (Robinson et al., 2010). The screening criteria for differentially expressed lncRNAs were |logFC|>1 and FDR<0.05.

### Real-Time Quantitative PCR

Total RNA was isolated from patient tissues in three groups using TRIzol reagent (Shanghai Biological Engineering Co., Ltd., Shanghai, China). Extracted total RNA (1 μl) was used for quality identification. When the OD260/280 value of the RNA sample was between 1.8 and 2.0, the sample was considered to be of high purity. The genome removal DNA reaction system included gDNA Eraser, 5x gDNA Eraser Buffer, RNA, and RNase Free dH2O. The reverse transcription system included PrimeScript RT Enzyme Mix I, RT Prime Mix, 5xPrimeScript Buffer 2, and RNase Free dH2O. Primer sequences used for RT-qPCR are listed in Table 1. TB Green™ Premix Ex Taq™ II kit (Takara, Shiga, Japan) and Pikoreal96 fluorescence quantitative PCR instrument (Thermo Fisher Scientific) for RT-qPCR. GAPDH was used as an internal reference gene, and the relative expression of each gene was displayed by the result of 2^−ΔΔCT^. The experiment for each group was repeated three times.

**TABLE 1:** Primer sequences used for RT-qPCR.

lncRNA	Forward primer	Reverse primer
GAPDH	GAG​TCA​ACG​GAT​TTG​GTC​GT	GAC​AAG​CTT​CCC​GTT​CTC​AG
lncRNA H19	ACC​ACT​GCA​CTA​CCT​GAC​TC	CCG​CAG​GGG​GTG​GCC​ATG​AA
LINC00895	AAG​AGG​ACA​CAA​CAA​GGC​ACC​ATC	GGA​AGT​CCA​AGA​TCA​AGG​CAC​CTG
lnc-SRGAP2C-16	AGT​GGA​GAT​TTC​AGC​CGC​TTT​GAG	AGT​TGA​ACG​CAC​ACA​TCA​CAA​AGG
lnc-B3GALT2-1	TGG​TGA​CTG​AAG​CAG​AGA​TTG​GTG	TCT​CTC​CTC​TTC​CTA​GCC​TTG​GTG
lnc-HLA-C-2	AGC​CAT​CTT​CCC​AGT​CCA​CCA​TC	CCA​CAG​CTC​CGA​TGA​CCA​CAA​C
lnc-APOC1-1	CCC​ATT​CCC​CGA​ACG​AAT​AAA​CCC	TCA​GAC​CAC​CTT​AGT​CCC​TTT​CCC

### Bioinformatic Analysis

We used two methods to predict targeted mRNAs: co-location and co-expression target mRNA prediction. The first method was to use the complementary pairing relationship between lncRNAs and known mRNAs and the location of lncRNAs on the chromosome relative to the mRNAs to predict target mRNAs. The second method was through correlation analysis of lncRNA and mRNA expression using the Pearson correlation coefficient method. The target mRNAs with Cor>0.99 were selected. We drew the interaction networks of lncRNA-mRNA pairs using Cytoscape software (v. 3.7.2).

GeneCards is an integrative database that provides comprehensive information on all annotated and predicted human genes (Barshir et al., 2021). LncSEA is an extensive human lncRNA sets resource and enrichment analysis platform (Chen et al., 2021). We attempted to find the gene symbols (e.g., H19, lnc-SRGAP2C-16) of the differentially expressed lncRNAs obtained by RNA-seq (e.g., NONHSAG007409.2, NONHSAG002625.2) through the GeneCard database, then imported them into the LncSEA website to conduct functional enrichment analysis.

Gene Ontology (GO) analysis and pathway analysis were used to determine the roles of target mRNAs of differentially expressed lncRNAs in the development of GC. We uploaded all target mRNAs into the Database for Annotation, Visualization, and Integrated Discovery (DAVID) for annotation and functional analysis. GO terms with a *p* value < 0.05 were selected. The top 10 enriched GO terms associated with the targeted mRNAs were presented. Kyoto Encyclopedia of Genes and Genomes (KEGG) pathway analysis was also performed to identify the significant pathways of the target genes.

### Statistics

In this study, GraphPad Prism 6.01 software was used for data processing, statistical analysis, and graphing. The data in this study conform to a normal distribution. We used Student’s t-test for comparison between the two groups. Statistical significance was set at *p* < 0.05.

## Results

### Screening of Differentially Expressed lncRNAs

We collected 45 clinical samples from the chronic non-atrophic gastritis group, gastric mucosal intraepithelial neoplasia group, and GC group and selected five high-quality samples from each group according to the quality control standards. The quality inspection results of the 15 samples are listed in Supplementary Table S1.

**FIGURE 1:**
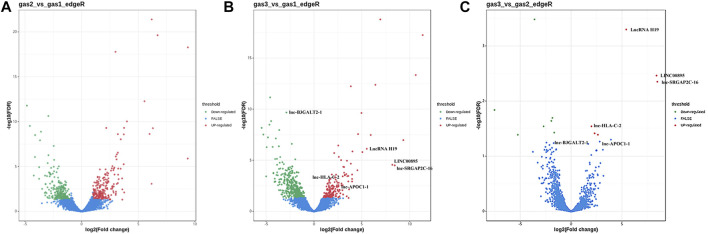
The volcano figures of upregulated and downregulated lncRNAs. **(A)** The differential lncRNAs between gastritis group (gas1) and gastric precancerous lesion group (gas2). **(B)** The differential lncRNAs between gastritis group (gas1) and gastric cancer group (gas3). **(C)** The differential lncRNAs between gastric precancerous lesion group (gas2) and gastric cancer group (gas3).

After the samples were sequenced, we used edgeR to analyze the difference in FPKM of lncRNA transcripts between samples according to the screening criteria of differentially expressed genes (|logFC|>1, FDR<0.05). The volcano figures display the upregulated and downregulated lncRNAs (Figure 1). Using the gastritis group (gas1) as the control group and GC group (gas3) as the experimental group for comparison, we found that a total of 666 lncRNAs with differential expression were identified, of which 188 were upregulated and 478 were downregulated (Supplementary Table S2). Using the gastric precancerous lesion group (gas2) as the control group and GC group (gas3) as the experimental group, we discovered that a total of 13 lncRNAs with differential expression were screened, including six with elevated expression levels and seven with decreased expression levels (Supplementary Table S3). Considering the gastritis group (gas1) as the control group and the gastric precancerous lesion group (gas2) as the experimental group, a total of 507 differential lncRNAs were screened, of which 236 increased and 271 decreased (Supplementary Table S4).

It was also found that six lncRNAs not only differentially expressed between gas1 and gas3, but also differentially expressed between gas2 and gas3 (Table 2). Among them, there were five upregulated lncRNAs, namely, lncRNA H19, LINC00895, lnc-SRGAP2C-16, lnc-HLA-C-2, and lnc-APOC1-1, and one downregulated lncRNA, lnc-B3GALT2-1.

**TABLE 2:** The LogFC and FDR of six differentially expressed lncRNAs between the gastritis group (gas1) and the gastric cancer group (gas3) as well as between the gastric precancerous lesions group (gas2) and the gastric cancer group (gas3).

lncRNA	gas1 and gas3		Gas2 and gas3	
	LogFC	FDR	LogFC	FDR
lncRNA H19	5.5199968785742	7.64E-07	5.41606758341622	0.000502885561838305
LINC00895	8.18159196198404	2.75992E-05	8.38571224191254	0.00345535762972322
lnc-SRGAP2C-16	8.43804447367164	3.15351E-05	8.47127707185439	0.004456797733884
lnc-HLA-C-2	1.73575759860498	0.000669082643182244	1.998919205213	0.0287657243650488
lnc-APOC1-1	1.95442546138273	0.00524648132938214	2.63051911970753	0.0409010786302443
lnc-B3GALT2-1	−2.87368305530439	2.1373E-10	−1.93861796711571	0.0229344445543282

### Expression Verification of Differentially Expressed lncRNAs

**FIGURE 2:**
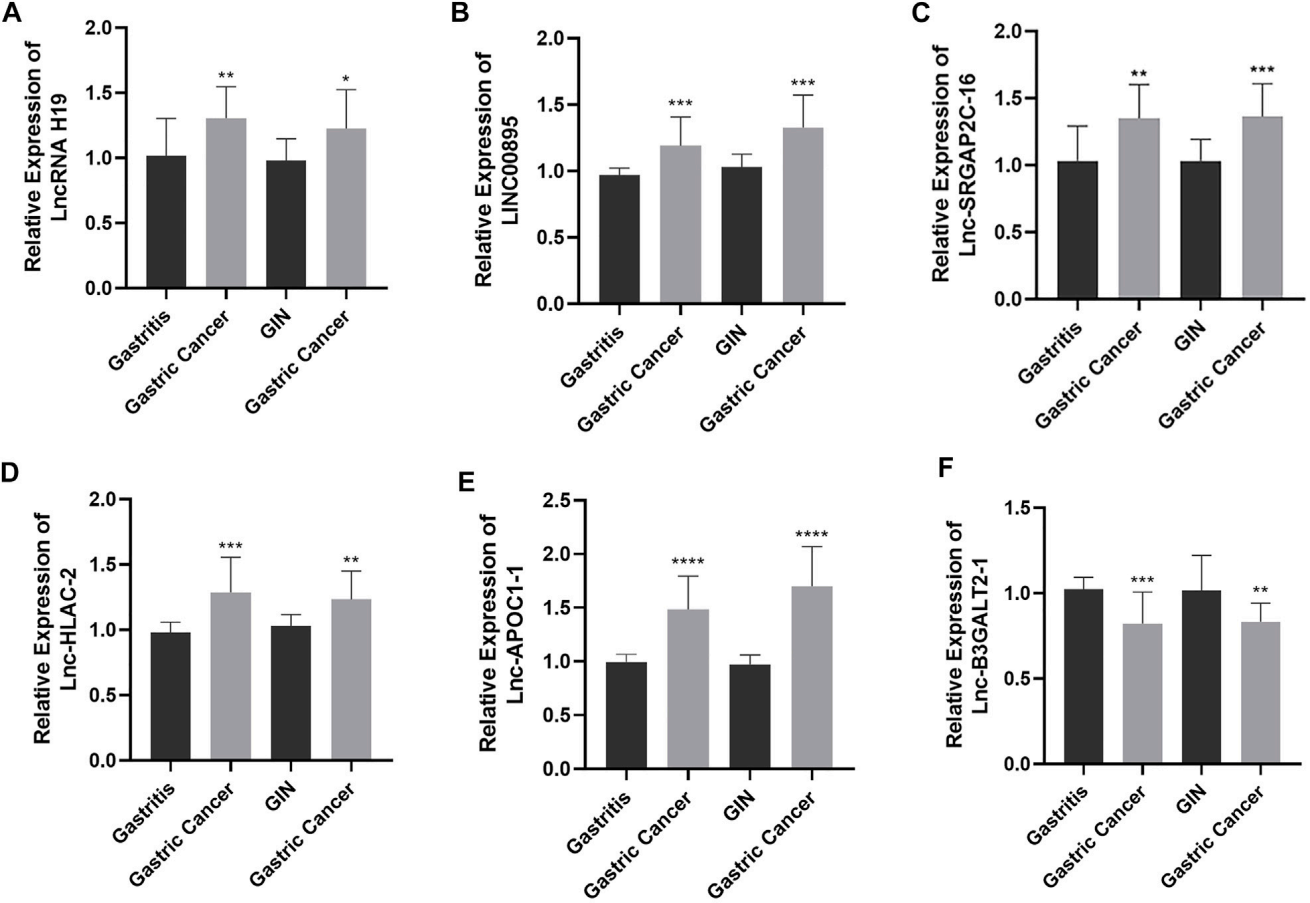
The expression levels of lncRNA H19 **(A)**, LINC00895 **(B)**, lnc-SRGAP2C-16 **(C)**, lnc-HLA-C-2 **(D)**, lnc-APOC1-1 **(E)**, and lnc-B3GALT2-1 **(F)** in chronic non-atrophic gastritis (Gastritis) vs. gastric cancer (Gastric Cancer) tissues and in gastric mucosal low-grade/high-grade intraepithelial neoplasia (GIN) vs. gastric cancer (Gastric Cancer) tissues.

To verify the expression of the six differentially expressed lncRNAs identified by sequencing analysis, we used RT-qPCR to evaluate the expression levels of lncRNA H19, LINC00895, lnc-SRGAP2C-16, lnc-HLA-C-2, lnc-APOC1-1, and lnc-B3GALT2-1 in 15 samples of chronic non-atrophic gastritis (gastritis), gastric mucosal low-grade/high-grade intraepithelial neoplasia (GIN), and GC (Gastric Cancer) tissues. The results are shown in Figure 2. Compared with the chronic non-atrophic gastritis group, the expression of lncRNA H19 (*n* = 10, *p* < 0.01, Figure 2A), LINC00895 (*n* = 10, *p* < 0.001, Figure 2B), lnc-SRGAP2C-16 (*n* = 10, *p* < 0.01, Figure 2C), lnc-HLA-C-2 (*n* = 10, *p* < 0.001, Figure 2D), and lnc-APOC1-1 (*n* = 10, *p* < 0.0001, Figure 2E) was significantly increased in the GC group, whereas lnc-B3GALT2-1 expression (*n* = 10, *p* < 0.001, Figure 2F) was significantly decreased. Compared with the gastric mucosal low-grade/high-grade intraepithelial neoplasia group, the expression level of lncRNA H19 (*n* = 10, *p* < 0.05, Figure 2A), LINC00895 (*n* = 10, *p* < 0.001, Figure 2B), lnc-SRGAP2C-16 (*N* = 10, *p* < 0.001, Figure 2C), lnc-HLA-C-2 (*n* = 10, *p* < 0.01, Figure 2D), lnc-APOC1-1 (n = 10, *p* < 0.0001, Figure 2E) in the GC group was significantly upregulated, while the expression level of lnc-B3GALT2-1 (*n* = 10, *p* < 0.01, Figure 2F) was significantly downregulated. The above results were consistent with the expression of the six differentially expressed lncRNAs obtained by sequencing.

### Functional Enrichment Analysis of Differentially Expressed lncRNAs

We conducted enrichment analysis for all the differentially expressed lncRNAs with corresponding gene symbols in GeneCard using LncSEA. Although the number of lncRNAs involved was limited, significant functional enrichment sets were still found (Table 3). H19 and IFNG-AS1 might be associated with ulcerative colitis (Cao et al., 2016; Fournet et al., 2001). Gallbladder cancer development might be related to H19 and FAM30A (Liu et al., 2016; Peng et al., 2017). H19 is involved in the invasion, migration, epithelial-mesenchymal transition (EMT), and metastasis of various cancers, such as hepatic cancer, pancreatic cancer, and gastric cancer (Supplementary Table S5). LINC00857 and LINC00261 are also related to cancer invasion and migration (Wang et al., 2016; Fan et al., 2016). lncRNAs are crucial in some essential epigenetic regulation processes, such as DNA methylation. Many studies have experimentally supported the associations between lncRNA H19 and DNA methylation, including demethylation, hypermethylation, and differential methylation (Supplementary Table S6). DLEU1 is linked to demethylation in leukemia, and LINC00857 is correlated to demethylation in NSCLC (Garding et al., 2013; Horie et al., 2017). Previous research found that TERC is associated with hypermethylation in breast cancer and differential methylation in dyskeratosis congenita (Gadalla et al., 2012; Heng et al., 2017). IGF2BP3 and IGF2BP2 are two classes of RNA binding proteins of the differentially expressed lncRNAs. Many short or small ORFs (sORFs/smORFs) can encode small peptides in the body. It has been found that eight differentially expressed lncRNAs have the smORF named lincRNA.

**TABLE 3:** Enrichment analysis of differentially expressed lncRNAs through LncSEA.

Enrichment set	Class	Sub_Class	Count	*p*-value	FDR	LncRNA	References
Ulcerative_colitis	Disease	LncRNADisease2.0	2	2.29E-05	0.00882	H19	Fournet et al. (2001), Cao et al. (2016)
						IFNG-AS1	
Gallbladder_cancer_	Disease	EVLncRNAs	2	0.000114	0.0299	H19	Liu et al. (2016), Peng et al. (2017)
						FAM30A	
invasion	Cancer Hallmark	—	3	0.00421	0.0112	H19^a^	Fan et al. (2016), Wang et al. (2016)
						LINC00857	
						LINC00261	
migration	Cancer Hallmark	—	3	0.00542	0.0112	H19^a^	Fan et al. (2016), Wang et al. (2016)
						LINC00857	
						LINC00261	
EMT	Cancer Hallmark	—	2	0.00611	0.0112	H19^a^	Fan et al. (2016)
						LINC00261	
metastasis	Cancer Hallmark	—	2	0.0064	0.0112	H19^a^	Fan et al. (2016)
						LINC00261	
demethylation	Methylation Pattern	—	3	1.38E-05	6.90E-05	H19^a^;	Garding et al. (2013), Horie et al. (2017)
						DLEU1;	
						LINC00857	
hypermethylation	Methylation Pattern	—	2	0.00275	0.00673	H19^a^	Heng et al. (2017)
						TERC	
differential methylation	Methylation Pattern	—	2	0.00404	0.00673	H19^a^	Gadalla et al. (2012)
						TERC	
IGF2BP3	RNA Binding Protein	starBasev2.0	5	7.00E-05	0.00189	SNHG6	N/A^b^
						SNHG4	
						LINC00261	
						TERC	
						LINC00311	
IGF2BP2	RNA Binding Protein	starBasev2.0	5	0.00141	0.019	H19	N/A
						SNHG6	
						DLEU1	
						SNHG4	
						TERC	
lincRNA	SmORF	sor.forg	8	0.000222	0.00133	H19	N/A
						FAM30A	
						SNHG6	
						DLEU1	
						SNHG4	
						LINC02057	
						LINC00857	
						LINC00261	

a Details of H19 are provided in the Supplementary Table S5, S6

b N/A, not applicable.

### Relationship Between Differentially Expressed lncRNAs and mRNAs

We utilized the complementary pairing relationship and the position of the differentially expressed lncRNAs relative to the mRNAs on the chromosome to predict target mRNAs. We also predicted lncRNA-targeted genes by co-expression analysis. The Pearson correlation coefficient method was used to analyze the correlation between the differentially expressed lncRNAs and mRNAs. We found 278 target genes of differentially expressed lncRNAs between gas1 and gas3 after deleting duplicate genes (Supplementary Table S7). The target genes of differentially expressed lncRNAs between gas2 and gas3 were only identified by co-expression analysis, with a total of 54 genes (Supplementary Table S8). Furthermore, we identified 196 target genes of differentially expressed lncRNAs between gas1 and gas2 (Supplementary Table S9).

**FIGURE 3:**
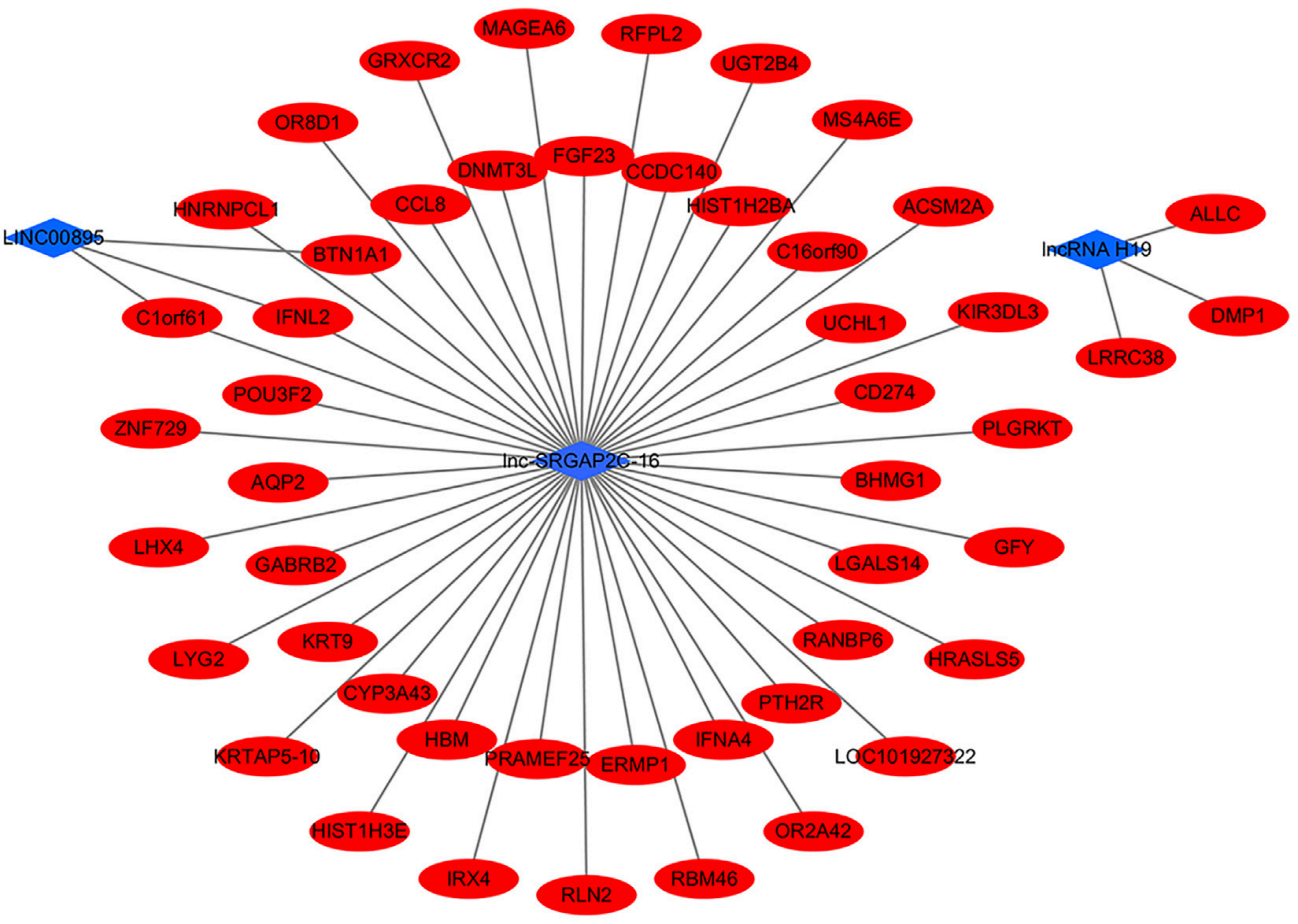
The interaction networks of differentially expressed lncRNAs (lncRNA H19, LINC00895, lnc-SRGAP2C-16) and target mRNAs.

Furthermore, we found that three of the six co-expressed differentially expressed lncRNAs had targeting correlation with mRNAs, namely lncRNA H19, LINC00895, lnc-SRGAP2C-16 (Figure 3). From the network, LINC00895 and lnc-SRGAP2C-16 had 3 same target mRNAs (BTN1A1, C1orf61, and IFNL2). lnc-SRGAP2C-16 had 46 target mRNAs, indicating that lnc-SRGAP2C-16 might play an important role in the occurrence and development of GC. This provides us with new insights for further exploring the biological mechanisms of lncRNA H19, LINC00895, and lnc-SRGAP2C-16 in the process of chronic non-atrophic gastritis and gastric mucosal low-grade or high-grade intraepithelial neoplasia to GC.

### Functional Enrichment Analysis of Target mRNAs

**FIGURE 4:**
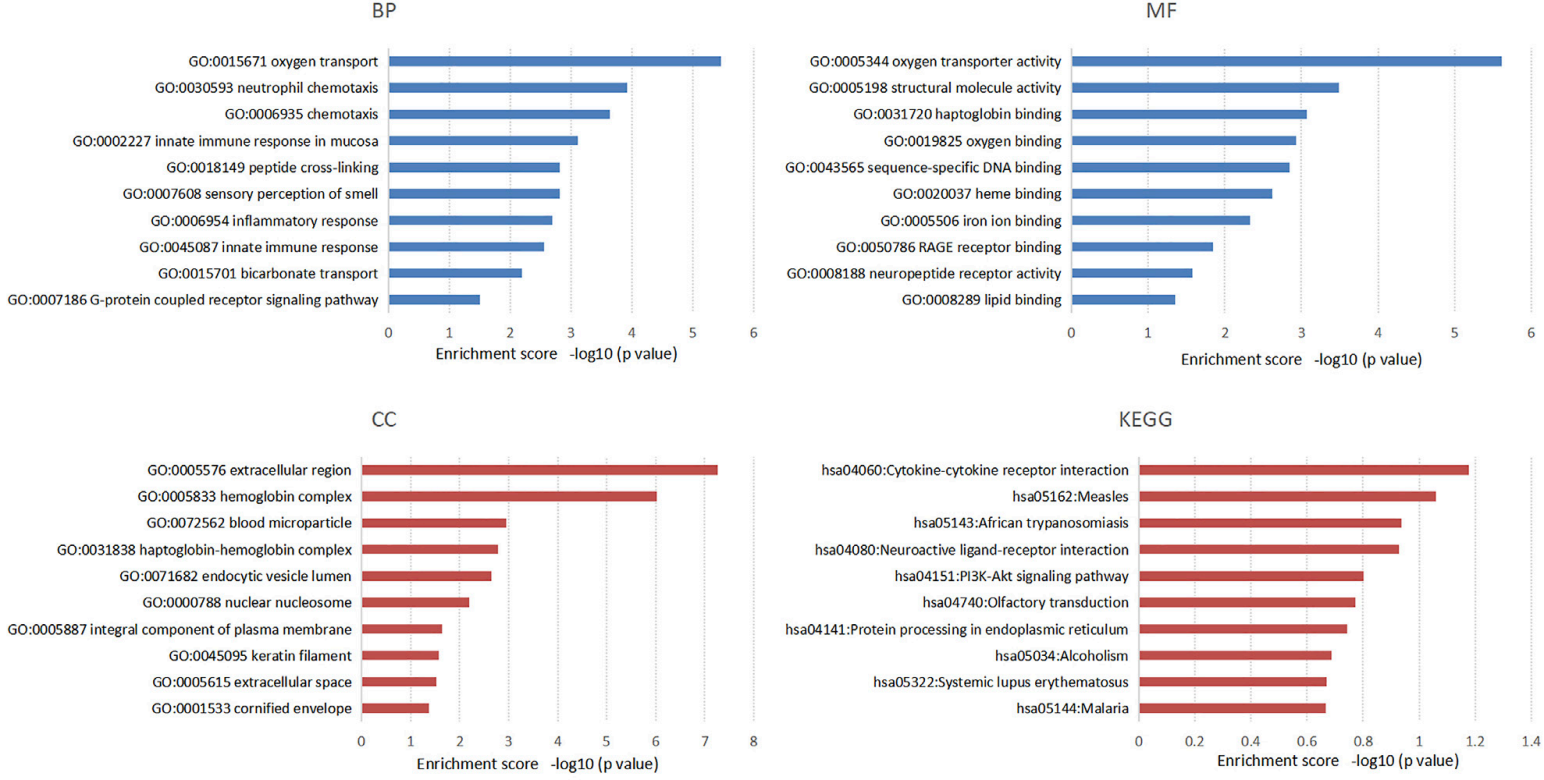
The top 10 enriched items of differential lncRNAs targeted mRNAs in the process of chronic non-atrophic gastritis and gastric mucosal intraepithelial neoplasia to gastric cancer in GO and KEGG enrichment analysis.

We also performed functional enrichment analysis on all the target mRNAs of differentially expressed lncRNAs. The enriched items and pathways of the differentially expressed lncRNA-targeted mRNAs are shown in Figure 4. The top 10 enriched items of targeted mRNAs in biological processes (BP) were oxygen transport, neutrophil chemotaxis, chemotaxis, innate immune response in mucosa, peptide cross-linking, sensory perception of smell, inflammatory response, innate immune response, bicarbonate transport, and G-protein coupled receptor signaling pathway. Furthermore, certain important GO functions might be related to oxygen transporter activity, structural molecule activity, haptoglobin binding, oxygen binding, sequence-specific DNA binding, heme binding, iron ion binding, RAGE receptor binding, neuropeptide receptor activity, and lipid binding in molecular function (MF). In addition, the extracellular region, hemoglobin complex, blood microparticle, haptoglobin-hemoglobin complex, endocytic vesicle lumen, nuclear nucleosome, integral component of plasma membrane, keratin filament, extracellular space, and cornified envelope were significant components of cellular components (CC). However, we found no significant KEGG pathways related to the target genes through the DAVID database.

## Discussion

Numerous studies have compared the gene expression profiles between tumor and normal tissues and found that various lncRNAs were differentially expressed in tumors, which may become potential tumor markers and targets for tumor intervention, providing new ideas for tumor diagnosis and treatment (Gu et al., 2015; Renganathan and Felley-Bosco, 2017). One study screened differential genes from GC and its adjacent normal tissues by microarray analysis and discovered that the expression levels of 33 lncRNAs were significantly different. The researchers further verified that the expression levels of HIF1A-AS1 and PVT1 were significantly downregulated in GC tissues, while the expression levels of CBR3-AS1 and UCA1 were significantly upregulated (Gao et al., 2015). Zhang et al. quantitatively evaluated the lncRNAs in GC cell lines and plasma and found that the expression of five lncRNAs in cancer cells was significantly increased, including TINCR (Zhang et al., 2017). More and more lncRNAs were found to act as carcinogens to promote the occurrence and development of GC.

In this study, we used RNA-seq technology to perform quantitative differential lncRNA expression analysis in the development of GC. We selected five high-quality samples from the chronic non-atrophic gastritis, gastric mucosal intraepithelial neoplasia, and GC groups for the construction of lncRNA libraries. We found that, compared with the chronic non-atrophic gastritis group, the GC group showed a total of 666 differential lncRNAs. Compared with the gastric mucosa low-grade/high-grade intraepithelial neoplasia group, the GC group showed a total of 13 differential lncRNAs. Taken together, there were 6 differential lncRNAs (lncRNA H19, LINC00895, lnc-SRGAP2C-16, lnc-HLA-C-2, lnc-APOC1-1, and lnc-B3GALT2-1) that were co-expressed in the same direction in the above two comparison results. Subsequently, we verified the difference in lncRNA expression levels between groups through RT-qPCR, which was consistent with the sequencing results, suggesting that the progression of chronic non-atrophic gastritis and gastric mucosal low-grade or high-grade intraepithelial neoplasia to GC was associated with the expression of the above six differentially expressed lncRNAs.

LncRNA H19, a star molecule in GC research, has already been reported to have carcinogenic mechanisms. Researchers analyzed the expression of lncRNAs in GC and normal tissues, indicating that H19 expression increased most significantly among 71 upregulated lncRNAs, approximately 9 times higher than that in normal tissues (Song et al., 2013). Yang et al. also found that the expression level of H19 in various GC cell lines and tissues was significantly higher than that in normal controls. Abnormal expression of H19 promoted the proliferation of AGS GC cell lines, while silencing H19 induced cell apoptosis. Mechanically, upregulated H19 participates in tumorigenesis by inhibiting p53 activation in GC (Yang et al., 2012). Zhuang et al. found that H19 predominantly targeted RUNX1 through miR-675 to regulate GC cell proliferation, providing a potential target for GC treatment (Zhuang et al., 2014). It has been reported that miR-141 binds to H19 to inhibit H19-induced proliferation and invasion. Furthermore, miR-141 and H19 could directly affect each other, and also competitively bind to each other’s target genes to affect the development of GC (Zhou et al., 2015). According to our verified experiments of differential lncRNAs, the expression level of lncRNA H19 in the GC group was significantly higher than that in the chronic non-atrophic gastritis group, which was consistent with the results of previous studies. In addition, our sequencing results also showed that LINC00895, lnc-SRGAP2C-16, lnc-HLA-C-2, and lnc-APOC1-1 were significantly increased in the GC compared to that of the chronic non-atrophic gastritis and gastric mucosal intraepithelial neoplasia groups, while lnc-B3GALT2-1 expression decreased significantly. However, currently, only LINC00895 has been found to be a high-frequency integration site of the HPV gene, which is related to HPV carcinogenesis (Yang-Chun et al., 2020). Thus, the mechanism of LINC00895 in GC requires further exploration. Notably, six lncRNAs co-expressed in the process of chronic non-atrophic gastritis and gastric mucosal low-grade or high-grade intraepithelial neoplasia to GC, and the expression levels were significantly different, indicating that the six differentially expressed lncRNAs might achieve the purpose of early GC diagnosis. In the future, we need to further investigate the six lncRNAs in body fluids to provide a new method for early GC diagnosis.

Through the LncSEA database, we directedly performed functional enrichment analysis for differentially expressed lncRNAs. In addition to H19, other differentially expressed lncRNAs are involved in disease occurrence, cancer invasion, metastasis, EMT, and methylation. However, the number of gene symbols of the differentially expressed lncRNAs found by GeneCard and the number of these lncRNAs involved in the LncSEA functional analysis were limited. Therefore, the results obtained by direct enrichment analysis of lncRNAs need further exploration. We are still a long way from unraveling the mystery of lncRNAs.

In addition, lncRNAs can bind to mRNAs and exhibit a wide range of biological functions. For example, lnc-LBCS directly interacts with hnRNPK and forms a complex with hnRNPK and AR mRNA to inhibit AR translation efficiency and cancer progression in castrated prostate cancer research (Gu et al., 2019). In short, analyzing the co-expression relationship of lncRNA-mRNA and identifying potential target genes of lncRNAs will help predict the function of lncRNAs. In this study, we found targeting correlations between three differentially expressed lncRNAs and mRNAs using the Pearson correlation coefficient method. The function of lncRNAs could be predicted through their target genes. It has been reported that BTN1A1 expression was essential for the regulation and secretion of milk fat droplets. Mechanistically, BTN1A1 might act as a structural protein or signal receptor by binding to xanthine dehydrogenase/oxidase (Ogg et al., 2004). C1orf61 acted as a tumor activator in hepatocellular carcinoma tumorigenesis and regulated diverse genes related to cell growth, migration, invasion and epithelial-mesenchymal transition (EMT) (Yu et al., 2021). At present, there is a lack of research on the mechanism of the three lncRNAs and target mRNAs in the tumorigenesis of GC and a lack of *in vivo* and *in vitro* experiments for verification. Therefore, we will focus on the underlying mechanism in our future research.

In previous studies, researchers focused on the expression of lncRNAs between GC and adjacent chronic non-atrophic gastritis but lacked attention to the differential lncRNAs between the gastric mucosa low-grade/high-grade intraepithelial neoplasia and GC. Therefore, we added the gastric mucosal low-grade/high-grade intraepithelial neoplasia group to discover the same differentially expressed lncRNAs in the three groups in this study. However, we only found differentially expressed lncRNAs during the development of GC. We should continue to expand the sample size to further analyze the specificity and sensitivity of six differential lncRNAs as markers for monitoring tumor progression and their correlation with clinicopathological parameters. Moreover, we should conduct long-term follow-up of patients and analyze the relationship between differentially expressed lncRNAs and survival. Subsequent research is expected to further explore the specific mechanisms of six lncRNAs in the development of GC and investigate their effects on the biological behaviors of GC cells, such as proliferation, invasion, induction of cell apoptosis, and EMT through *in vivo* and *in vitro* experiments, which will enrich the evidence of lncRNAs as biomarkers for early GC diagnosis.

## Data Availability

The datasets presented in this study can be found in online repositories. The names of the repository and accession number can be found below: CNGB Sequence Archive (CNSA) (Guo et al., 2020) of China National GeneBank DataBase (CNGBdb) (Chen et al., 2020) with accession number CNP0002510.

## Supplementary Material

Supplementary Table 1 The quality inspection results and reads quality of the 15 samples.

Supplementary Table 2 The differentially expressed lncRNAs between the chronic non-atrophic gastritis and GC groups.

Supplementary Table 3 The differentially expressed lncRNAs between the gastric mucosal intraepithelial neoplasia and GC groups.

Supplementary Table 4 The differentially expressed lncRNAs between the chronic non-atrophic gastritis and gastric mucosal intraepithelial neoplasia groups.

Supplementary Table 5 The association between H19 and various cancers in invasion, migration, EMT, and metastasis through LncSEA.

Supplementary Table 6 The association between H19 and Methylation Pattern through LncSEA.

Supplementary Table 7 The target genes of differentially expressed lncRNAs between the chronic non-atrophic gastritis and GC groups.

Supplementary Table 8 The target genes of differentially expressed lncRNAs between the gastric mucosal intraepithelial neoplasia and GC groups.

Supplementary Table 9 The target genes of differentially expressed lncRNAs between the chronic non-atrophic gastritis and gastric mucosal intraepithelial neoplasia groups.

Supplementary Table 10 The download links of softwares used in screening the differentially expressed lncRNAs.
